# Trends in long COVID among US adults, 2022–2024

**DOI:** 10.3389/fpubh.2026.1809635

**Published:** 2026-04-20

**Authors:** Xiaoli Jia, Jingjing Wang, Xiaomeng Cui, Ting Li, Yan Tian, Rui Lu, Yindi Tian, Xin Shen, Shuangsuo Dang, Wenjun Wang

**Affiliations:** 1 Department of Infectious Diseases, The Second Affiliated Hospital of Xi’an Jiaotong University, Xi’an, China; 2 Institute of Infectious Diseases, The Second Affiliated Hospital of Xi’an Jiaotong University, Xi’an, China; 3 Department of Pediatrics, The Second Affiliated Hospital of Xi’an Jiaotong University, Xi’an, China; 4 Medical Record Center, Shaanxi Provincial People’s Hospital, Xi’an, China

**Keywords:** COVID, long COVID, National Health Interview Survey, SARS-CoV-2, socioeconomic, trends

## Abstract

**Importance:**

Long COVID poses a significant public health challenge. However, population-level trends and associated factors in the general US population during the post-pandemic era are not fully characterized.

**Objective:**

To analyze trends in long COVID prevalence among US adults from 2022 to 2024, identify associated demographic and socioeconomic factors, and assess its impact on daily activities.

**Design, setting and participants:**

We analyzed data from three cycles (2022–2024) of the National Health Interview Survey, a repeated cross-sectional, nationally representative survey. COVID-19 infection, long COVID, and daily activity limitation were determined through participant self-report. Daily activity limitation was assessed in 2023–2024. Multivariable Poisson regression identified factors associated with: (1) long COVID history and (2) significant activity limitation among those with current symptoms. All analyses incorporated survey weights to produce nationally representative estimates. Data analysis was conducted in October 2025.

**Results:**

The study included 88,731 adults (median age 47 years; 51.4% female and 48.6% male; 61.7% non-Hispanic White, 17.6% Hispanic, 11.8% non-Hispanic Black, and 8.9% non-Hispanic other). In the overall population, the prevalence of ever long COVID increased from 7.0% (95% CI, 6.6–7.3%) in 2022 to 8.4% (95% CI, 8.0–8.8%) in 2023, plateauing at 8.3% (95% CI, 7.9–8.7%) in 2024, while for current long COVID the prevalence remained stable (3.4% [95% CI, 3.1–3.6%] in 2022, 3.6% [95% CI, 3.3–3.9%] in 2023, and 3.3 [95% CI, 3.1–3.6%] in 2024). Among adults with prior COVID-19 infection, prevalence of both ever and current long COVID declined significantly, from 17.7 to 13.7% and from 8.6 to 5.5%, respectively. Long COVID was more common among women, adults in middle age (35–64 years), Hispanic or non-Hispanic White individuals, those who were widowed/separated/divorced, people with lower educational attainment, and individuals with incomes below the federal poverty threshold. Among those with current long COVID, 19.8% reported significant activity limitation, and this limitation was more common among older adults and individuals with lower incomes.

**Conclusions and relevance:**

From 2022 to 2024, long COVID continued to impose a substantial public health burden, with clear demographic and socioeconomic disparities. These findings underscore the necessity for continued surveillance and targeted support for high-risk groups.

## Introduction

Long COVID is defined as the persistence of symptoms for 3 months or longer following acute SARS-CoV-2 infection (1, 2). Common manifestations include fatigue, shortness of breath, cognitive dysfunction, and other symptoms, which can lead to prolonged disability and diminished quality of life (1, 2). Despite numerous clinical trials, effective treatments for long COVID remain elusive. Emerging evidence underscores the substantial public health burden posed by long COVID, with millions worldwide experiencing persistent health impairment and reduced work capacity (3).

Recent national estimates indicate that long COVID remains prevalent among US adults, although its population-level trends in the post-pandemic period are not yet well established. In 2022, the prevalence of long COVID was 3.4% among US adults based on data from the National Health Interview Survey (NHIS), a national representative survey (4). By 2023, approximately 7.0% of US adults reported having ever experienced long COVID (4, 5). As SARS-CoV-2 has continued to evolve and population immunity has become widespread, the clinical presentation and epidemiological patterns of acute COVID-19 infections have shifted significantly. In this evolving landscape, it has been hypothesized that the burden of long COVID may show different trends compared to earlier periods of the pandemic (6). A rapid survey reported a decrease in long COVID prevalence from 7.5 to 6.0% among US adults between mid-2022 and mid-2023 (7). However, up-to-date data of higher-quality are needed to confirm this trend and characterize recent changes in long COVID prevalence.

Beyond its prevalence, previous studies have reported that approximately one-quarter of adults with long COVID experience significant limitations in daily activities (7). However, it has remained unclear whether the prevalence or severity of these activity limitations has changed over time, and which risk factors are associated with them. Women, middle-aged adults, socioeconomic disadvantaged individuals, and individuals with preexisting conditions were previously reported as risk factors for long COVID (3, 8, 9). These factors represent key demographic and socioeconomic characteristics that may influence both susceptibility to long COVID and the likelihood or reporting persistent symptoms, thereby shaping the observed heterogeneity in long COVID burden across the population. However, most existing studies examining risk factors for long COVID have relied on convenience samples, localized cohorts or electronic health records (3, 8, 9). Such data sources tend to capture healthcare-seeking individuals, who may be more likely to have severe acute infections or preexisting conditions. As a result, the risk factors identified in these studies may not be fully generalizable to the general population as they excluded the infected ones who did not seek health service.

To address these gaps in population-based research on long COVID, this study examines nationwide trends in long COVID prevalence, identifies the demographic and socioeconomic factors associated with its occurrence, and assesses its impact on daily activities, using a national repeat cross-sectional survey of US adults from 2022 to 2024. Critically, this study assessed cumulative prevalence (ever long COVID), active prevalence (current long COVID), and the functional burden (activity limitations), providing a multi-dimensional view of the public health impact.

## Methods

### Study population

The NHIS is an ongoing nationally representative household survey conducted by the National Center for Health Statistics (NCHS) at the Centers for Disease Control and Prevention (10). It assesses the health status of the US civilian noninstitutionalized population through continuous data collection. For this study, we analyzed three independent cycles of NHIS data from 2022, 2023 and 2024, each comprising distinct participant samples. The NCHS Institutional Review Board approved the survey protocols, and verbal consent was obtained from all participants. Given the de-identified and publicly available nature of the data, this study was exempt from ethical review by Xi’an Jiaotong University. Response rates for the Sample Adult component were 47.7% in 2022, 47.0% in 2023, and 47.9 in 2024 (11–13).

### Data collection

Data were collected by trained interviewers primarily through in-person household interviews, with telephone follow-ups when necessary. Demographic and socioeconomic variables included age, sex, race/ethnicity (non-Hispanic White, non-Hispanic Black, non-Hispanic other, or Hispanic), marital status (married/unmarried couple, single, or widowed/separated/divorced), educational attainment (less than high school, high school graduate/some college, or college graduate), household income (below or at/above the federal poverty threshold), and health insurance coverage (yes or no). These variables were assessed using standardized NHIS questionnaires.

### Outcomes

The primary outcomes were the prevalence of ever long COVID and the prevalence of current long COVID. Ever long COVID was based on a “yes” response to the survey question, “Did you have any symptoms lasting 3 months or longer that you did not have prior to having COVID-19?” among those who reported receiving either a positive test or a doctor’s diagnosis of COVID-19 and were symptomatic. Participants with asymptomatic infections or uncertain infection status were excluded from the long COVID definition, while those with multiple infections were included if they reported qualifying symptoms. Among participants who reported ever long COVID, those with ongoing symptoms at the time of the survey were classified as having current long COVID (Supplementary method).

The secondary outcome, assessed in the 2023 and 2024 cycles, was the impact of current long COVID on daily activities. Respondents with current long COVID were asked to indicate to what extent their daily activities were limited: “not at all”, “a little”, or “a lot” (Supplementary method). COVID-19 infection, long COVID, and daily activity limitation were determined through participant self-report following survey instructions.

### Statistical analysis

The prevalences of ever long COVID and current long COVID were calculated separately for each survey year using data from 2022, 2023, and 2024 NHIS cycles among adults aged 18 years and older. Prevalence was calculated for the overall adult population and across predefined demographic and socioeconomic subgroups, including age (18–34, 35–49, 50–64, and ≥65 years), sex, race/ethnicity, marital status, educational attainment, household income and health insurance coverage (insured or uninsured). To assess changes across survey years, linear regression models were employed to estimate the absolute difference in prevalence. These prevalence estimates and temporal changes were also calculated among adults reporting prior COVID-19 infection, using the same analytic approach.

Factors associated with long COVID were examined among adults reporting prior COVID-19 infection. Poisson regression was employed to assess the prevalence ratios for long COVID across various demographic and socioeconomic characteristics, including age group, sex, race/ethnicity, marital status, education, household income, and health insurance coverage. Poisson regression was also employed to examine factors associated with significant daily activity limitation among adults with current long COVID. All aforementioned variables were adjusted.

Given minimal missing data (<1.2%), imputation was not performed. All analyses incorporated NHIS sampling weights to ensure nationally representative estimates, accounting for the complex survey design, nonresponse, and calibration adjustments. Taylor series linearization with finite population correction was used to compute 95% confidence intervals (CIs). A two-tailed *p*-value <0.05 was considered statistically significant. However, due to the exploratory nature of secondary analyses and the risk of type I error from multiple comparisons, these findings should be interpreted cautiously. All analyses were conducted using StataSE 15 (StataCorp). Data analysis was conducted in October 2025. The study adheres to STROBE guidelines.

## Results

### Population characteristics

Across the 2022–2024 NHIS cycles, 89,802 adults aged ≥18 years were surveyed. After excluding 1,071 participants with missing information on COVID-19 illness or long COVID, 88,731 participants were included for analysis. These participants had a median age of 47 years (interquartile range, 32–63 years), with 48.6% of them being male. Regarding race and ethnicity, 61.7% were non-Hispanic, 17.6% Hispanic, 11.8% non-Hispanic Black and 8.9% non-Hispanic other (Supplementary Table S1).

Among the 88,731 participants, 43,981 (weighted percentage of 51.8%) reported prior COVID-19 infection; their characteristics are shown in Supplementary Table S2. In 2022, 39.4% (95% CI, 38.7–40.1%) of US adults reported prior COVID-19 infection after being weighted to the national population. This percentage rose to 55.5% (95% CI, 54.7–56.2%) in 2023 and further to 60.4% (95% CI, 59.7–61.1%) in 2024 (Supplementary Figure S1). This increasing trend was observed across almost all demographic and socioeconomic subgroups (Supplementary Figure S1).

### Trends in prevalence of long COVID among overall adult population

**Figure 1:**
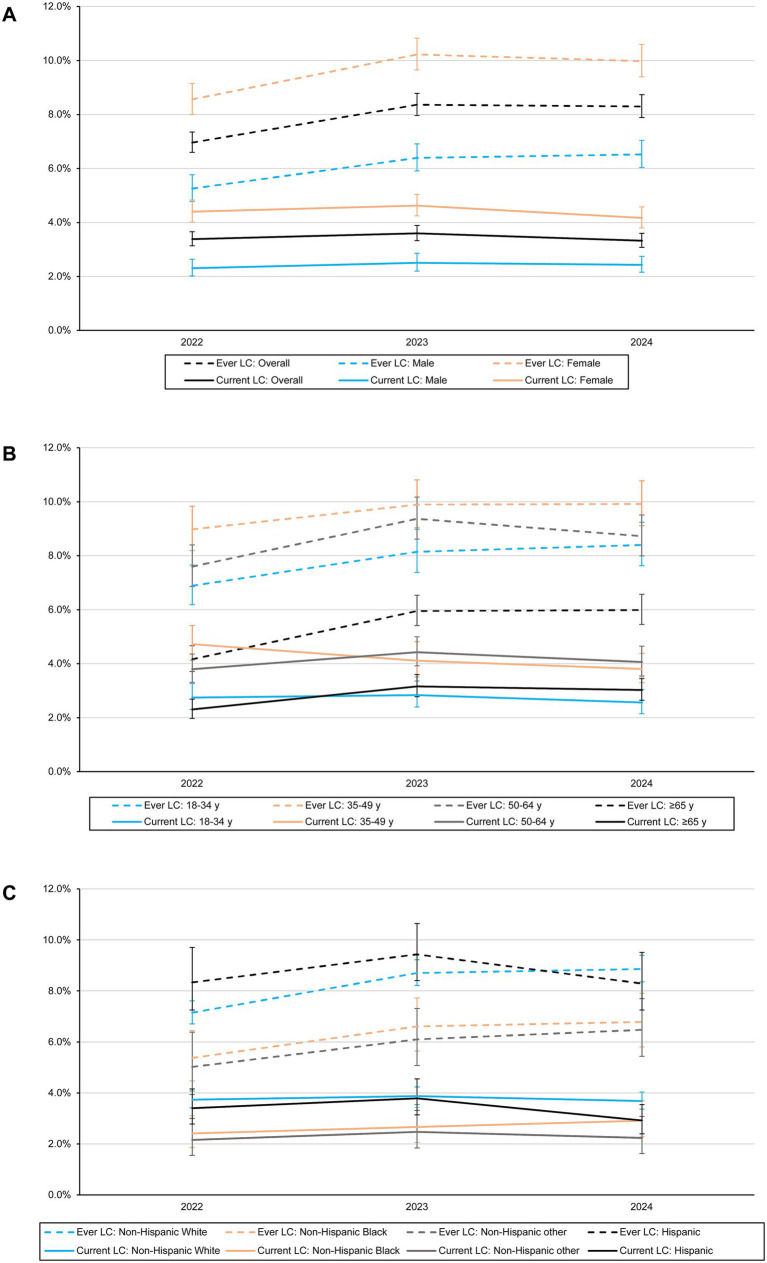
Prevalence of ever and current long COVID-19 among overall US adults **(A)** and by sex **(A)**, age **(B)**, and race/ethnicity **(C)** from 2022 to 2024. LC, long COVID. Error bars indicate 95% CIs.

The prevalence of ever long COVID increased from 7.0% in 2022 (95% CI, 6.6–7.3%) to 8.4% in 2023 (95% CI, 8.0–8.8%; 2022 vs. 2023: *p* < 0.001), then plateaued at 8.3% in 2024 (95% CI, 7.9–8.7%; 2023 vs. 2024: *p* = 0.817) among overall US adult population, irrespective of prior COVID-19 infection (Figure 1A). Significant increases between 2022 and 2024 were observed across most demographic and socioeconomic subgroups, including all age groups except those aged 35–49 years, both men and women, non-Hispanic White adults, single or coupled adults, and those with higher education or higher income (Figures 1A–C; Table 1; Supplementary Table S3).

**Table 1:** Prevalence of ever long COVID and current long COVID among US adults, The National Health Interview Survey, 2022–2024.

Characteristic	Prevalence, % (95% CI)^a^			Changes across years, % (95% CI)^a^					
	2022	2023	2024	2022 vs 2023^b^	*p* value^b^	2023 vs 2024^b^	*p* value^b^	2022 vs 2024^b^	*p* value^b^
Ever long COVID									
Long COVID reporters/participants, No.	1,797/27,388	2,398/29,123	2,627/32,220						
Overall	7.0 (6.6–7.3)	8.4 (8.0–8.8)	8.3 (7.9–8.7)	1.4 (0.9–1.9)	<0.001	−0.1 (−0.6–0.5)	0.817	1.3 (0.8–1.9)	<0.001
Age, year									
18–34	6.9 (6.2–7.7)	8.1 (7.4–9.0)	8.4 (7.6–9.2)	1.3 (0.2–2.3)	0.023	0.3 (−0.9–1.4)	0.653	1.5 (0.4–2.6)	0.006
35–49	9.0 (8.2–9.8)	9.9 (9.0–10.8)	9.9 (9.1–10.8)	0.9 (−0.2–2.1)	0.120	0.0 (−1.1–1.2)	0.964	0.9 (−0.2–2.1)	0.112
50–64	7.6 (6.9–8.4)	9.4 (8.6–10.2)	8.7 (8.0–9.5)	1.8 (0.7–2.9)	0.001	−0.6 (−1.7–0.4)	0.224	1.1 (0.1–2.2)	0.034
≥65	4.2 (3.7–4.7)	6.0 (5.4–6.5)	6.0 (5.4–6.6)	1.8 (1.1–2.5)	<0.001	0.0 (−0.7–0.8)	0.929	1.8 (1.1–2.5)	<0.001
Sex									
Male	5.3 (4.8–5.8)	6.4 (5.9–6.9)	6.5 (6.0–7.0)	1.1 (0.4–1.8)	0.001	0.1 (−0.6–0.8)	0.711	1.3 (0.6–2.0)	<0.001
Female	8.6 (8.0–9.1)	10.2 (9.6–10.8)	10.0 (9.4–10.6)	1.7 (0.9–2.4)	<0.001	−0.2 (−1.0–0.6)	0.550	1.4 (0.6–2.2)	0.001
Race/ethnicity^c^									
Non-Hispanic White	7.1 (6.7–7.6)	8.7 (8.2–9.2)	8.9 (8.4–9.4)	1.6 (0.9–2.2)	<0.001	0.2 (−0.5–0.8)	0.652	1.7 (1.0–2.4)	<0.001
Non-Hispanic Black	5.4 (4.5–6.4)	6.6 (5.6–7.7)	6.8 (5.8–7.9)	1.2 (−0.2–2.6)	0.082	0.2 (−1.3–1.6)	0.813	1.4 (0.0–2.8)	0.052
Non-Hispanic other	5.0 (3.9–6.4)	6.1 (5.1–7.3)	6.5 (5.4–7.7)	1.1 (−0.5–2.6)	0.169	0.4 (−1.1–1.9)	0.619	1.5 (0.0–3.0)	0.057
Hispanic	8.3 (7.3–9.5)	9.4 (8.4–10.5)	8.3 (7.4–9.3)	1.1 (−0.3–2.5)	0.130	−1.1 (−2.5–0.2)	0.103	0.0 (−1.4–1.3)	0.945
Marital status									
Married/unmarried couple	7.3 (6.9–7.8)	8.8 (8.3–9.4)	8.8 (8.3–9.4)	1.5 (0.8–2.2)	<0.001	0.0 (−0.7–0.7)	0.999	1.5 (0.8–2.2)	<0.001
Single	5.8 (5.1–6.6)	7.2 (6.5–8.1)	7.2 (6.4–8.1)	1.4 (0.3–2.5)	0.012	0.0 (−1.2–1.1)	0.977	1.4 (0.2–2.5)	0.018
Widowed/separated/divorced	7.1 (6.4–7.9)	8.7 (7.9–9.6)	8.2 (7.4–9.0)	1.6 (0.5–2.7)	0.006	−0.5 (−1.6–0.6)	0.384	1.1 (0.0–2.2)	0.054
Highest education level									
Less than high school	6.2 (5.0–7.6)	7.1 (6.0–8.5)	6.9 (5.7–8.3)	1.0 (−0.9–2.8)	0.301	−0.2 (−1.9–1.5)	0.809	0.8 (−1.0–2.6)	0.408
High school graduate and some college	7.5 (7.0–8.0)	9.0 (8.4–9.6)	8.8 (8.3–9.4)	1.5 (0.8–2.2)	<0.001	−0.2 (−0.9–0.5)	0.607	1.3 (0.5–2.1)	0.001
College graduate	6.3 (5.8–6.9)	7.7 (7.1–8.3)	7.9 (7.3–8.5)	1.4 (0.6–2.1)	0.001	0.2 (−0.6–1.0)	0.620	1.6 (0.8–2.3)	<0.001
Income									
At or above poverty threshold	6.9 (6.6–7.4)	8.2 (7.8–8.7)	8.3 (7.9–8.7)	1.3 (0.7–1.8)	<0.001	0.1 (−0.5–0.6)	0.820	1.4 (0.8–1.9)	<0.001
Below poverty threshold	7.1 (6.0–8.3)	9.5 (8.2–11.0)	8.3 (7.2–9.6)	2.4 (0.7–4.2)	0.007	−1.2 (−3.0–0.6)	0.187	1.2 (−0.3–2.8)	0.125
Health insurance									
Yes	7.1 (6.7–7.5)	8.4 (8.0–8.8)	8.3 (7.9–8.8)	1.3 (0.8–1.9)	<0.001	−0.1 (−0.6–0.5)	0.826	1.3 (0.7–1.9)	<0.001
No	5.9 (4.8–7.3)	7.8 (6.4–9.4)	7.9 (6.5–9.4)	1.9 (−0.1–3.8)	0.059	0.1 (−1.9–2.1)	0.926	2.0 (0.0–3.9)	0.046
Current long COVID									
Long COVID reporters/participants, No.	919/27,388	1,063/28,060	1,106/31,114						
Overall	3.4 (3.1–3.6)	3.6 (3.3–3.9)	3.3 (3.1–3.6)	0.2 (−0.2–0.6)	0.261	−0.3 (−0.6–0.1)	0.135	−0.1 (−0.4–0.3)	0.747
Age, year									
18–34	2.7 (2.3–3.3)	2.8 (2.4–3.4)	2.6 (2.1–3.0)	0.1 (−0.6–0.7)	0.775	−0.3 (−0.9–0.4)	0.387	−0.2 (−0.8–0.4)	0.568
35–49	4.7 (4.1–5.4)	4.1 (3.5–4.8)	3.8 (3.3–4.4)	−0.6 (−1.5–0.3)	0.174	−0.3 (−1.1–0.5)	0.459	−0.9 (−1.7–−0.1)	0.028
50–64	3.8 (3.3–4.3)	4.4 (3.9–5.0)	4.1 (3.5–4.6)	0.6 (−0.1–1.4)	0.100	−0.4 (−1.1–0.4)	0.347	0.3 (−0.5–1.0)	0.494
≥65	2.3 (2.0–2.7)	3.2 (2.8–3.6)	3.0 (2.6–3.4)	0.9 (0.3–1.4)	0.003	−0.1 (−0.7–0.4)	0.622	0.7 (0.2–1.3)	0.008
Sex									
Male	2.3 (2.0–2.6)	2.5 (2.2–2.9)	2.4 (2.2–2.7)	0.2 (−0.3–0.7)	0.384	−0.1 (−0.5–0.4)	0.733	0.1 (−0.3–0.6)	0.565
Female	4.4 (4.0–4.8)	4.6 (4.2–5.0)	4.2 (3.8–4.6)	0.2 (−0.3–0.8)	0.439	−0.5 (−1.0–0.1)	0.092	−0.2 (−0.8–0.3)	0.398
Race/ethnicity^c^									
Non-Hispanic White	3.7 (3.4–4.1)	3.9 (3.5–4.2)	3.7 (3.4–4.0)	0.1 (−0.3–0.6)	0.561	−0.2 (−0.7–0.3)	0.420	0.0 (−0.5–0.4)	0.842
Non-Hispanic Black	2.4 (1.9–3.1)	2.7 (2.1–3.4)	2.9 (2.3–3.7)	0.3 (−0.7–1.2)	0.593	0.2 (−0.7–1.2)	0.617	0.5 (−0.5–1.5)	0.309
Non-Hispanic other	2.2 (1.6–3.0)	2.5 (1.8–3.3)	2.2 (1.6–3.1)	0.3 (−0.7–1.3)	0.541	−0.2 (−1.2–0.7)	0.635	0.1 (−0.9–1.1)	0.881
Hispanic	3.4 (2.8–4.2)	3.8 (3.1–4.6)	2.9 (2.4–3.5)	0.4 (−0.5–1.2)	0.385	−0.9 (−1.7–0.0)	0.043	−0.5 (−1.3–0.3)	0.250
Marital status									
Married/unmarried couple	3.7 (3.3–4.0)	3.9 (3.5–4.3)	3.5 (3.2–3.9)	0.2 (−0.3–0.7)	0.411	−0.4 (−0.8–0.1)	0.156	−0.1 (−0.6–0.3)	0.566
Single	2.2 (1.8–2.7)	2.6 (2.1–3.1)	2.5 (2.1–3.0)	0.4 (−0.3–1.0)	0.270	0.0 (−0.7–0.6)	0.901	0.3 (−0.3–0.9)	0.336
Widowed/separated/divorced	3.9 (3.3–4.5)	4.4 (3.8–5.0)	4.0 (3.4–4.6)	0.5 (−0.3–1.3)	0.207	−0.4 (−1.2–0.4)	0.340	0.1 (−0.7–1.0)	0.781
Highest education level									
Less than high school	2.2 (1.6–3.0)	3.8 (2.9–4.9)	2.5 (1.8–3.4)	1.6 (0.3–2.8)	0.013	−1.3 (−2.5–−0.1)	0.032	0.3 (−0.7–1.3)	0.613
High school graduate and some college	3.8 (3.5–4.2)	3.9 (3.5–4.3)	3.6 (3.2–3.9)	0.0 (−0.5–0.6)	0.862	−0.3 (−0.8–0.2)	0.257	−0.2 (−0.8–0.3)	0.361
College graduate	3.0 (2.7–3.4)	3.1 (2.7–3.5)	3.1 (2.7–3.5)	0.0 (−0.5–0.6)	0.923	0.0 (−0.5–0.6)	0.883	0.1 (−0.5–0.6)	0.809
Income									
At or above poverty threshold	3.4 (3.1–3.7)	3.5 (3.2–3.8)	3.4 (3.1–3.7)	0.1 (−0.3–0.5)	0.496	−0.1 (−0.5–0.2)	0.474	0.0 (−0.4–0.4)	0.992
Below poverty threshold	3.4 (2.7–4.2)	4.3 (3.5–5.3)	2.8 (2.3–3.5)	0.9 (−0.3–2.0)	0.134	−1.4 (−2.5–−0.3)	0.010	−0.6 (−1.5–0.4)	0.227
Health insurance									
Yes	3.5 (3.2–3.8)	3.7 (3.4–4.0)	3.4 (3.1–3.7)	0.2 (−0.2–0.6)	0.340	−0.3 (−0.6–0.1)	0.154	−0.1 (−0.5–0.3)	0.681
No	2.4 (1.8–3.3)	2.7 (1.9–3.8)	2.6 (1.9–3.6)	0.3 (−0.9–1.4)	0.668	−0.1 (−1.3–1.2)	0.898	0.2 (−1.0–1.3)	0.762

^a^All estimates, except the numbers of participants and long COVID reporters, were weighted to reflect population estimates. ^b^Linear regression was employed to estimate the absolute difference in prevalences. ^c^Race and Hispanic ethnicity were self-reported and classified based on the 1997 Office of Management and Budget Standards.

By contrast, the prevalence of current long COVID remained stable during the same period, at 3.4% (95% CI, 3.1–3.6%) in 2022, 3.6% (95% CI, 3.3–3.9%) in 2023, and 3.3% (95% CI, 3.1–3.6%) in 2024 (Figure 1A). Subgroup patterns were generally consistent across demographic and socioeconomic categories. However, from 2022 to 2024 a decline was observed among adults aged 35–49 years, whereas an increase exhibited among those aged 65 years and older (Figures 1A–C; Table 1; Supplementary Table S3).

### Trends in prevalence of long COVID among adults with prior COVID-19 infection

**Figure 2:**
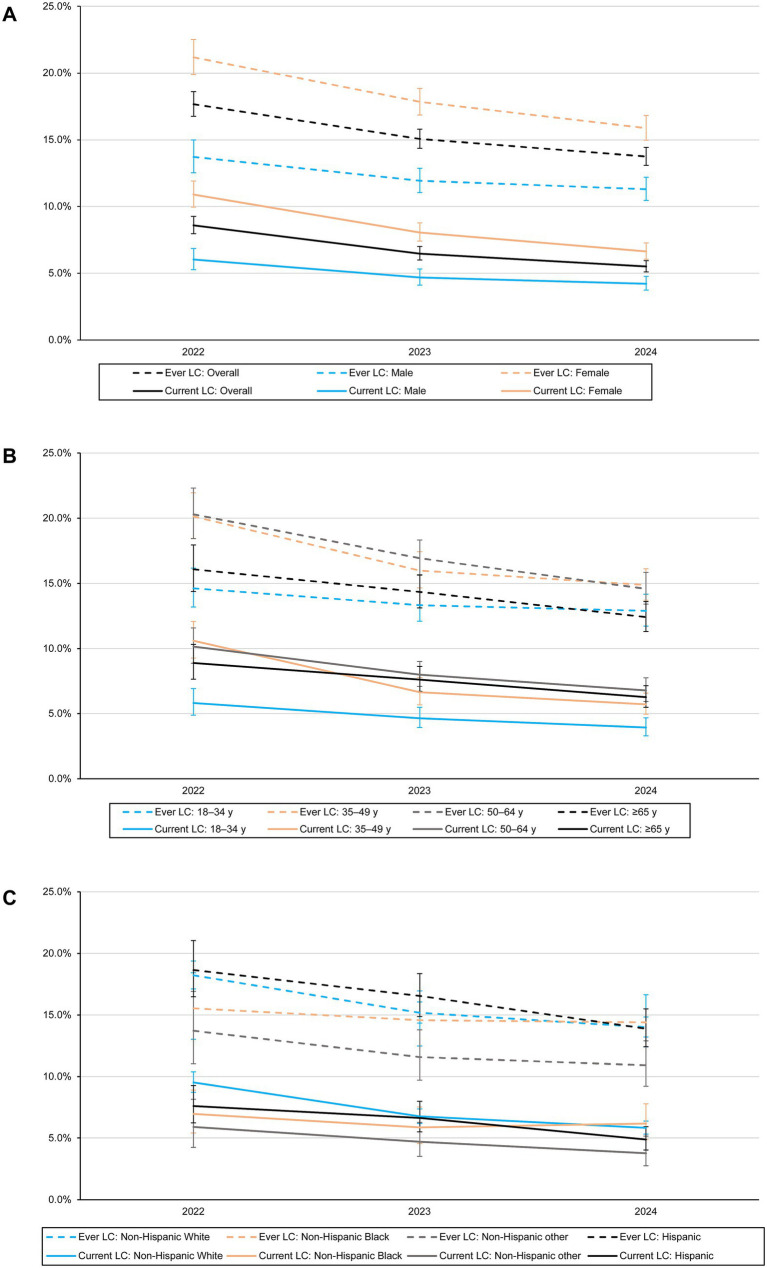
Prevalence of ever and current long COVID-19 among US adults with COVID-19 history **(A)** and by sex **(A)**, age **(B)**, and race/ethnicity **(C)** from 2022 to 2024. LC, long COVID. Error bars indicate 95% CIs.

Among adults with prior COVID-19 infection, the prevalence of ever long COVID decreased from 17.7% (95% CI, 16.8–18.6%) in 2022 to 15.1% (14.4–15.8%) in 2023 and further to 13.7% (13.1–14.4%) in 2024 (Figure 2A). The prevalence of current long COVID also decreased over time, from 8.6% (95% CI, 8.0–9.3%) in 2022 to 6.5% (95% CI, 6.0–7.0%) in 2023 and 5.5% (95% CI, 5.1–5.9%) in 2024 (Figure 2A). The declining trend was observed across most demographic and socioeconomic subgroups. However, no clear decline was observed among non-Hispanic Black, non-Hispanic other, single individuals, those with less than high school education, and those without health insurance (Figures 2A–C; Supplementary Table S4).

### Factors associated with ever long COVID

**Figure 3:**
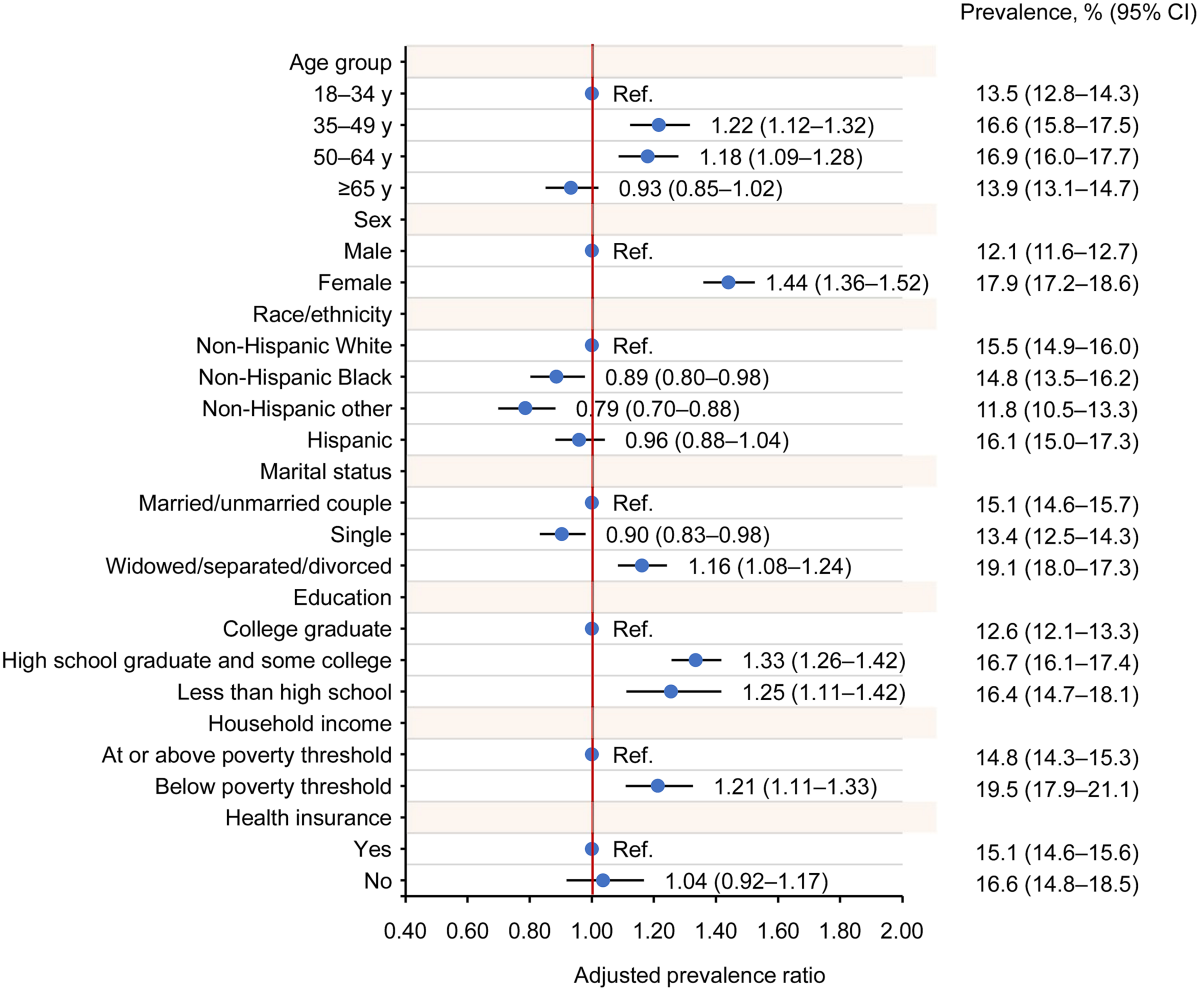
Factors associated with long COVID among US adults with COVID-19 history. Multivariate Poisson regression with survey weights was used to adjust for age, sex, race/ethnicity, marital status, education, household income, and health insurance. The prevalence and 95% CI were weighted to reflect population estimates.

Multiple demographic and socioeconomic factors were found to be associated with ever long COVID, including sex, age, race/ethnicity, marital status, education, and household income (Figure 3). Among adults with prior COVID-19 infection, females exhibited a higher prevalence of ever long COVID than males (17.9% vs. 12.1%; adjusted prevalence ratio [aPR], 1.44 [95% CI, 1.36–1.52]) during 2022–2024. Using adults aged 18–34 years as the reference group, ever long COVID was more prevalent among those aged 35–49 years (16.6% vs. 13.5%; aPR, 1.22 [95% CI, 1.12–1.32]) and 50–64 years (16.9% vs. 13.5%; aPR, 1.18 [95% CI, 1.09–1.28]), while the prevalence in those aged ≥65 years was comparable to those aged 18–34 years (13.9% vs. 13.5%; aPR, 0.93 [95% CI, 0.85–1.02]). By race/ethnicity, the prevalence of ever long COVID was lower among non-Hispanic Black adults (14.8%) and non-Hispanic adults of other races (11.8%) compared with non-Hispanic White adults (15.5%) (aPR, 0.89 [95% CI, 0.80–0.98], and 0.79 [95% CI, 0.70–0.88], respectively). Hispanic and non-Hispanic White exhibited similar prevalence (16.1% vs. 15.5%; aPR, 0.96 [95% CI, 0.88–1.04]). Ever long COVID was more prevalent among adults who were widowed, separated, or divorced, compared with those who were married or living with a partner (19.1% vs. 15.1%; aPR, 1.16 [95% CI, 1.08–1.24]). In contrast, the prevalence was lower among single adults (13.4% vs. 15.1%; aPR, 0.90 [95% CI, 0.83–0.98]). Compared with adult who had a college education, the prevalence was higher among those with less than a high school education (16.4% vs. 12.6%; aPR, 1.25 [95% CI, 1.11–1.42]) and those with a high school education (16.7% vs. 12.6%; aPR, 1.33 [95% CI, 1.26–1.42]). In addition, adults below the poverty threshold showed higher ever long COVID prevalence than those at or above the poverty threshold (19.5% vs. 15.1%; aPR, 1.21 [95% CI, 1.11–1.33]).

### Activity limitation associated with current long COVID

Among adults reporting current long COVID in the 2023 and 2024 survey cycles, 45.4% (95% CI, 42.9–47.9%) and 19.8% (95% CI, 18.0–21.7%) reported that their daily activities were limited “a little” and “a lot,” respectively (Table 2), with no significant changes from 2023 to 2024 (Supplementary Figure S2–S5). Multivariate analysis showed that adults of older age or lower household income were more likely to report “a lot” of limitation in their daily activities (Supplementary Figure S6).

**Table 2:** Percentage of US adults with activity limitation among those with current long COVID, The National Health Interview Survey, 2023–2024.

Characteristic	Long COVID, N (weighted %)^a^ (*N* = 2,163)	Daily activity limitation		
		A lot, weighted % (95% CI)^a^ (*N* = 466)	A little, weighted % (95% CI)^a^ (*N* = 973)	Not at all, weighted % (95% CI)^a^ (*N* = 724)
Overall	1,059 (100)	19.8 (18.0–21.7)	45.4 (42.9–47.9)	34.8 (32.3–37.3)
Age group, year				
18–34	359 (22.7)	12.6 (9.4–16.8)	46.8 (40.9–52.8)	40.6 (34.9–46.6)
35–49	512 (28.0)	18.4 (14.8–22.6)	46.2 (41.3–51.2)	35.4 (30.7–40.4)
50–64	638 (29.0)	23.0 (19.2–27.2)	43.9 (39.1–48.7)	33.2 (28.9–37.7)
≥65	653 (20.4)	25.3 (21.5–29.6)	44.9 (39.9–49.9)	29.8 (25.9–34.1)
Sex				
Male	722 (34.7)	19.1 (16.0–22.6)	39.8 (35.4–44.3)	41.1 (36.8–45.7)
Female	1,440 (65.3)	20.2 (18.0–22.6)	48.3 (45.2–51.5)	31.5 (28.6–34.5)
Race/ethnicity^b^				
Non-Hispanic White	1,545 (67.3)	20.0 (17.8–22.5)	43.8 (40.9–46.9)	36.1 (33.3–39.1)
Non-Hispanic Black	185 (9.3)	22.3 (16.0–30.1)	47.5 (38.9–56.4)	30.2 (22.5–39.2)
Non-Hispanic other	128 (6.0)	16.5 (11.1–23.9)	54.3 (43.7–64.6)	29.1 (20.7–39.3)
Hispanic	305 (17.3)	18.8 (14.6–23.8)	47.1 (40.9–53.4)	34.1 (28.0–40.8)
Marital status				
Married/unmarried couple	1,107 (64.1)	18.4 (16.1–21.0)	46.6 (43.3–49.9)	35.0 (31.8–38.3)
Single	361 (17.5)	16.1 (12.0–21.4)	47.2 (40.4–54.1)	36.7 (30.2–43.6)
Widowed/separated/divorced	646 (18.4)	25.8 (21.7–30.4)	41.5 (36.9–46.3)	32.7 (28.4–37.3)
Highest education level				
Less than high school	162 (9.6)	24.5 (17.8–32.7)	45.5 (36.8–54.4)	30.1 (21.7–40.0)
High school graduate and some college	1,274 (60.6)	20.9 (18.6–23.5)	44.0 (40.6–47.5)	35.0 (31.9–38.3)
College graduate	717 (29.8)	15.3 (12.5–18.7)	48.5 (44.3–52.7)	36.2 (32.2–40.4)
Income				
At or above poverty threshold	1,912 (89.6)	18.2 (16.3–20.2)	45.5 (42.7–48.1)	36.4 (33.7–39.2)
Below poverty threshold	251 (10.4)	33.9 (26.7–42.0)	45.3 (37.7–53.2)	20.8 (14.5–28.9)
Health insurance				
Yes	1,050 (94.1)	19.5 (17.7–21.4)	45.2 (42.6–47.8)	35.1 (32.7–37.9)
No	110 (5.9)	24.5 (16.1–35.5)	47.4 (36.1–59.0)	28.1 (19.3–38.9)

^a^All percentage estimates were weighted and numbers were unweighted participant numbers. ^b^Race and Hispanic ethnicity were self-reported and classified based on the 1997 Office of Management and Budget Standards.

## Discussion

Using nationally representative NHIS data, we found a divergence between trends in ever and current long COVID among overall US adult population. The prevalence of ever long COVID increased from 2022 (7.0%) to 2023 (8.4%) and plateaued in 2024 (8.3%), whereas the prevalence of current long COVID showed little change over the same period (3.4% in 2022, 3.6% in 2023, and 3.3% in 2024). Despite this stabilization, an estimated 3.3% of US adults, representing millions of individuals, reported current long COVID in 2024, underscoring its persistent burden on patients, clinicians, and healthcare systems.

The stable trend of current long COVID observed in this study differs from estimates based on the US Household Pulse Survey (HPS), another nationally representative source tracking long COVID among US adults (7). HPS showed that the prevalence decreased from 7.5 to 6.0% from mid-2022 to mid-2023. However, it should be noted that HPS has considerably lower response rates (3.9–7.0%) and relies on non-probability sampling conducted via mobile phone and email (14). These features raise concerns regarding potential selection biases and the reliability of its prevalence estimates. Indeed, estimates of current long COVID in HPS were nearly twice as high as those based on other national surveys, including NHIS and Behavioral Risk Factor Surveillance System (4, 15). In contrast, NHIS employs rigorous sampling methods and collects data through in-person interview. NHIS also applies robust weighting adjustments for nonresponse and achieves significantly higher response rates (47.0–47.9% during 2022–2024) (11–13). Together, these methodological strengths support NHIS as a more reliable source for tracking national trends in long COVID.

The prevalence of current long COVID was stable or declined across most subgroups, except for adults aged 65 years and older. In this age group, prevalence increased from 2.3% in 2022 to 3.2% in 2023 and then plateaued at 3.0% in 2024. Prior research indicates that older adults are more likely to experience prolonged COVID-19 symptoms with delayed recovery (16). Consistent with these findings, our analysis showed that older adults with current long COVID were more likely to report significant limitation in daily activities. These findings may explain the distinct prevalence trend of long COVID observed among older adults and highlight potential long-term demands for geriatric and chronic care services.

Unlike the overall population, the prevalence of current and ever long COVID among adults with prior COVID-19 infection decreased steadily from 2022 to 2024. This pattern does not contradict the stable trend observed in the overall population, as new COVID-19 infections continued to accumulate during this period, serving as an ongoing source of new long COVID cases (Supplementary Figure S1). As COVID-19 transitioned from a pandemic to an endemic phase, the incidence of new infections began to decline. Supplementary Figure S1 also suggests that the rate of cumulative infections slowed during 2023–2024 compared with 2022–2023. Therefore, as individuals with long COVID recover over time and fewer new cases emerge, the prevalence of current long COVID will decline in the future.

Population immunity, virus evolution, and antiviral treatments may contribute to the decline in long COVID prevalence among adults with prior COVID-19. Widespread vaccination and natural immunity from prior infections have enhanced population-level protection against severe and prolonged COVID-19 symptoms. Evidence from three meta-analyses highlights a dose-dependent protective effect of vaccines in reducing the risk of lingering symptoms (17–19). Notably, booster vaccination uptake among US adults surged from 25.7% in November 2021 to 63.4% by March 2022 (20), which significantly amplified the protective shield against long COVID. Additionally, the dominance of milder Omicron subvariants may further reduce long COVID risk. Studies revealed that compared with infections during the Delta wave, infections during the Omicron wave were associated with a 56–58% reduction in long COVID risk (21, 22). Advances in acute-phase treatments, such as antiviral therapies, have also played a pivotal role by curbing severe infections that often precede long COVID (23).

Even as long COVID prevalence among those with prior COVID-19 declines, the symptoms continue to disproportionately burden certain populations. While many previous studies focused on the healthcare-seeking population, this study identifies associated factors for long COVID among the general population with a history of COVID-19. Middle-aged adults (35–64 years), women, non-Hispanic White and Hispanic individuals, and socioeconomically disadvantaged groups seem to more likely to report long COVID. The observed inverted U-shaped age distribution, characterized by a peak in prevalence among middle-aged adults, is consistent with findings from both US and international cohorts (5, 9, 24). This distribution can be explained by several interrelated mechanisms. Older adults are less likely to recognize their symptoms as long COVID since they may misattribute their symptoms to age-related comorbidities. Meanwhile, the acute symptoms in younger adults are mostly mild and these individuals may be underrepresented due to lower symptom recognition (9).

In addition to age, sex disparities are also pronounced in long COVID prevalence, with women exhibiting nearly 1.5 times the prevalence observed in men. This disparity may reflect a complex interplay of biological, immunological and social factors. Biologically, women have a higher predisposition to autoimmune conditions, and sex hormones are known to modulate immune responses, potentially increasing vulnerability to post-viral sequelae (25, 26). Social factors such as female’s caregiving roles and differential symptom reporting may also play a role and warrant further investigation (27, 28). Additionally, our study found that, Black adults and non-Hispanic adults of other races reported a lower prevalence of long COVID compared to White individuals, consistent with findings from another population-based survey (5).

Widowed, separated, or divorced adults, those with lower educational attainment, and individuals with lower income experience a disproportionately higher prevalence of long COVID, since socioeconomic factors further amplify inequities (29–31). These disparities mirror the patterns observed during acute COVID and likely stem from structural barriers such as occupational exposures and limited healthcare access (30). Individuals in these groups often have higher baseline burden of chronic disease and fewer opportunities to take time off for rest and recovery, potentially prolonging symptoms and impairing resolution (32).

The functional consequences of long COVID are particularly concerning. Approximately 20% of adults with current long COVID reported significant activity limitations (“a lot”), and this percentage did not change much in 2023 (20.8%) and 2024 (18.8%). The prevalence of significant activity limitations increased with age, from 12.6% among adults aged 18–34 to 25.3% among those aged 65 and older, indicating the cumulative burden of comorbidities and age-related vulnerability. Socioeconomic disparities further exacerbate these challenges. Adults living below the poverty threshold are 1.8 times more likely to suffer from significant limitations, mirroring national trends in which disadvantaged populations bear a disproportionate burden of both acute and long-term COVID-19 outcomes. The functional impairments associated with long COVID can be profound. In one study, over half of affected individuals with long COVID reported moderate-to-severe functional deficits, comparable to the levels observed among patients with advanced cancer (33). The workforce consequences are similarly sobering. Estimates suggest that 18 to 26% of employed individuals with long COVID either reduced work hours or left jobs within a year of symptom onset (34). Such disruptions extend beyond individual health, potentially straining economic productivity and caregiving systems, particularly when working-age adults struggle to maintain employment or meet family responsibilities (35).

Our findings provide several public health and clinical implications. As COVID-19 shifts toward endemicity, long COVID remains a consequential post-acute sequela and requires coordinated efforts across healthcare and policy sectors. Continued population-level surveillance may be essential to monitoring the evolving patterns in long COVID prevalence as new variants and vaccination strategies emerge. Interventions may be tailored to address disparities, particularly among women, middle-aged adults, and socioeconomically disadvantaged populations.

Several limitations should be acknowledged. First, the reliance on self-reported COVID, long COVID, and activity limitations introduces potential recall bias, which may contribute to misclassification, particularly given symptom non-specificity and overlap with chronic conditions. While we used CDC’s definition of long COVID, its definition may vary to some extent among health organizations, researchers, and clinicians, depending on symptom duration (4 weeks to three or more months), required symptom count, and whether the cases are self-reported or clinically confirmed. These inconsistencies affect prevalence estimates. Second, the cross-sectional design precludes causal inference. In particular, socioeconomic variables such as marital status and income could be both risk factors and consequences of long COVID. The exclusion of institutionalized populations may underestimate the prevalence of long COVID. Third, residual confounding is possible due to lack of information on illness severity, timing of vaccination relative to infection, treatments during acute infection, symptom duration, reinfection, and underlying chronic diseases. Although NHIS collects vaccination status, the lack of data on vaccination timing relative to infection limited our ability to assess vaccine effectiveness in preventing long COVID. Fourth, activity limitation was assessed only in 2023–2024 and in some cases, it may reflect baseline disability rather than impairment specifically attributable to long COVID. In addition, NHIS collected limited information about function impairment. Future studies should assess both the type and extent of functional limitations more comprehensively. Fifth, due to the risk of type I error from multiple comparisons, results of subgroup analysis should be interpreted with caution.

In conclusion, this study provides critical insights into the trends of long COVID during 2022–2024 in the United States. Long COVID continues to exert a substantial public health burden. Its significant impact on daily activities underscores the need for continued surveillance, public health interventions, and healthcare support to mitigate the long-term consequences. Future research should prioritize longitudinal methodologies to elucidate risk factors, mechanisms, and long-term outcomes of long COVID, alongside evaluating the efficacy of preventive and therapeutic strategies.

## Data Availability

The datasets presented in this study can be found in online repositories. The names of the repository/repositories and accession number(s) can be found below: The dataset is freely available for download at the NHIS websites (https://www.cdc.gov/nchs/nhis/index.html).
