# Supplementary material for: Trends in long COVID among US adults, 2022–2024
Source: Front Public Health. 2026 Apr 20;14:1809635. doi: 10.3389/fpubh.2026.1809635 (PMC13136101; doi:10.3389/fpubh.2026.1809635)
Supplement: Supplementary file 1 [file Supplementary_file_1.docx]

**Supplementary Material**

Trends in Long COVID Among US Adults, 2022 to 2024.

**Supplementary method** Questionaries about COVID-19, long COVID, and the impact of long COVID on daily activities

**Table S1** Characteristics of US adults, 2022-2024

**Table S2** Characteristics of US adults with prior COVID-19, 2022-2024

**Table S3.** Relative prevalence change of ever long COVID and current long COVID among US adults, the National Health Interview Survey, 2022-2024

**Table S4.** Prevalence of ever long COVID and current long COVID among US adults with prior COVID-19, 2022-2024

**Figure S1** Cumulative infection rate of COVID-19 among overall US adults (A) and by sex (A), age (B), race/ethnicity (C), marital status (D), education (E), income (F), and insurance coverage (G) from 2022 to 2024.

**Figure S2** Daily activity limitation among US adults reporting current long COVID, 2023-2024

**Figure S3** Daily activity limitation by sex among US adults reporting current long COVID, 2023-2024

**Figure S4** Daily activity limitation by age among US adults reporting current long COVID, 2023-2024

**Figure S5** Daily activity limitation by race/ethnicity among US adults reporting current long COVID, 2023-2024

**Figure S6** Factors associated with significant activity limitation among US adults with current long COVID.

**Supplementary method**

**Questionaries about COVID-19, long COVID, and the impact of long COVID on daily activities**

**2022 cycle**

CVDDIAG_A: Has a doctor or other health professional ever told you that you had or likely had coronavirus or COVID-19?

POSTEST_A: Did you ever take a test that showed you had coronavirus or COVID-19? *(Testing includes antibody or blood tests as well as other forms of testing for COVID-19, such as a nasal swabbing or throat swabbing.)*

CVDSEV_A: How would you describe your coronavirus symptoms when they were at their worst? Would you say no symptoms, mild symptoms, moderate symptoms, or severe symptoms?

LONGCVD_A: Did you have any symptoms lasting 3 months or longer that you did not have prior to having coronavirus or COVID-19? *(Long term symptoms may include tiredness or fatigue, difficulty thinking, concentrating, forgetfulness or memory problems, sometimes referred to as "brain fog," difficulty breathing or shortness of breath, joint or muscle pain, fast-beating or pounding heart (also known as heart palpitations), chest pain, dizziness on standing, depression, anxiety or mood changes.)*

SYMPNOW_A: Do you have symptoms NOW?

**2023 cycle**

EVERCOVD_A: Have you ever had COVID-19? *(Include being told by a doctor or other health professional that you had or likely had COVID-19. Also include antibodies or blood tests as well as other forms of testing for COVID-19, such as a nasal swabbing or throat swabbing. Also include if you had close contact with someone who had COVID-19 and you had symptoms.)*

LONGCOVD1_A: Did you have any symptoms lasting 3 months or longer that you did not have prior to having COVID-19? *Long term symptoms may include tiredness or fatigue, difficulty thinking, concentrating, forgetfulness or memory problems, sometimes referred to as "brain fog," difficulty breathing or shortness of breath, joint or muscle pain, fast-beating or pounding heart (also known as heart palpitations), chest pain, dizziness on standing, ^menstrual changes to taste/smell, and inability to exercise.*

SYMPNOW1_A: Do you have symptoms NOW?

LCVDACT_A: How much do these long-term symptoms reduce your ability to carry out day-today activities compared with the time before you had COVID-19? Would you say not at all, a little, or a lot?

**2024 cycle**

EVERCOVD_A: Have you ever had COVID-19? *(Include being told by a doctor or other health professional that you had or likely had COVID-19. Also include antibodies or blood tests as well as other forms of testing for COVID-19, such as a nasal swabbing or throat swabbing. Also include if you had close contact with someone who had COVID-19 and you had symptoms.)*

LONGCOVD2_A: Did you have any symptoms lasting 3 months or longer that you did not have before having COVID-19? *(Long term symptoms may include tiredness or fatigue, difficulty thinking, concentrating, forgetfulness or memory problems, sometimes referred to as "brain fog," difficulty breathing or shortness of breath, joint or muscle pain, fast-beating or pounding heart (also known as heart palpitations), chest pain, dizziness on standing, smell or taste loss or alteration to smell or taste, ^menstrual and inability to exercise.)*

SYMPNOW1_A: Do you have symptoms NOW?

LCVDACT_A: How much do these long-term symptoms reduce your ability to carry out day-today activities compared with the time before you had COVID-19? Would you say not at all, a little, or a lot?

**Table S1. Characteristics of US adults, 2022-2024**

| **Characteristic** | **Raw No. (Weighted %)^a^** |
| --- | --- |
| No. of adults | 88,731 |
| Age, median (IQR), year | 47 (32–63) |
| Age group, y |  |
| 18–34 | 18,353 (28.9) |
| 35–49 | 19,619 (24.4) |
| 50–64 | 21,553 (24.0) |
| ≥65 | 29,110 (22.6) |
| Sex |  |
| Male | 40,472 (48.6) |
| Female | 48,246 (51.4) |
| Race/ethnicity^b^ |  |
| Non-Hispanic White | 58,806 (61.7) |
| Non-Hispanic Black | 9,479 (11.8) |
| Non-Hispanic other | 7,439 (8.9) |
| Hispanic | 13,007 (17.6) |
| Marital status |  |
| Married/unmarried couple | 45,087 (60.6) |
| Single | 18,381 (23.9) |
| Widowed/separated/divorced | 21,989 (15.5) |
| Highest education level |  |
| Less than high school | 7,476 (10.5) |
| High school graduate and some college | 47,260 (56.3) |
| College graduate | 33,578 (33.1) |
| Income |  |
| At or above poverty threshold | 79,537 (90.0) |
| Below poverty threshold | 9,194 (10.0) |
| Health insurance |  |
| Yes | 83,023 (92.0) |
| No | 5,612 (8.0) |

^a^ Percentages were weighted to reflect population estimates.

^b^ Race and Hispanic ethnicity were self-reported and classified based on the 1997 Office of Management and Budget Standards.

**Table S2. Characteristics of US adults with prior COVID-19, 2022-2024**

| **Characteristic** | **Raw No. (Weighted %)^a^** |
| --- | --- |
| No. of adults | 43,981 |
| Age, median (IQR), y | 47 (32-63) |
| Age group, y |  |
| 18–34 | 10,869 (32.3) |
| 35–49 | 11,408 (27.3) |
| 50–64 | 10,723 (23.5) |
| ≥65 | 10,941 (16.9) |
| Sex |  |
| Male | 19,369 (46.9) |
| Female | 24,606 (53.1) |
| Race/ethnicity^b^ |  |
| Non-Hispanic White | 29,449 (63.4) |
| Non-Hispanic Black | 3,820 (9.7) |
| Non-Hispanic other | 3,680 (8.5) |
| Hispanic | 7,032 (18.4) |
| Marital status |  |
| Married/unmarried couple | 24,560 (64.2) |
| Single | 8,996 (23.3) |
| Widowed/separated/divorced | 8,871 (12.4) |
| Highest education level |  |
| Less than high school | 2,875 (8.4) |
| High school graduate and some college | 22,378 (54.7) |
| College graduate | 18,554 (37.0) |
| Income |  |
| At or above poverty threshold | 40,281 (91.8) |
| Below poverty threshold | 3,700 (8.2) |
| Health insurance |  |
| Yes | 41,500 (93.3) |
| No | 2,439 (6.7) |

^a^ Percentages were weighted to reflect population estimates.

^b^ Race and Hispanic ethnicity were self-reported and classified based on the 1997 Office of Management and Budget Standards.

**Table S3. Relative prevalence change of ever long COVID and current long COVID among US adults, the National Health Interview Survey, 2022-2024**

|  | **2022-2023** | **2023-2024** | **2022-2024** |
| --- | --- | --- | --- |
| **Ever long COVID** |  |  |  |
| Overall | 20.1% | -0.7% | 19.2% |
| Age, year |  |  |  |
| 18–34 | 18.3% | 3.1% | 22.0% |
| 35–49 | 10.2% | 0.3% | 10.5% |
| 50–64 | 23.3% | -6.9% | 14.8% |
| ≥65 | 43.0% | 0.6% | 43.8% |
| Sex |  |  |  |
| Male | 21.6% | 2.1% | 24.1% |
| Female | 19.5% | -2.4% | 16.6% |
| Race/ethnicity |  |  |  |
| Non-Hispanic White | 21.8% | 1.8% | 24.0% |
| Non-Hispanic Black | 23.0% | 2.6% | 26.2% |
| Non-Hispanic other | 21.4% | 6.2% | 29.0% |
| Hispanic | 13.1% | -12.1% | -0.6% |
| Marital status |  |  |  |
| Married/unmarried couple | 20.4% | 0.0% | 20.4% |
| Single | 24.0% | -0.2% | 23.7% |
| Widowed/separated/divorced | 22.4% | -5.7% | 15.4% |
| Highest education level |  |  |  |
| Less than high school | 15.7% | -3.0% | 12.3% |
| High school graduate and some college | 20.1% | -2.2% | 17.5% |
| College graduate | 21.3% | 2.7% | 24.5% |
| Income |  |  |  |
| At or above poverty threshold | 18.5% | 0.8% | 19.4% |
| Below poverty threshold | 34.4% | -12.6% | 17.4% |
| Health insurance |  |  |  |
| Yes | 19.0% | -0.7% | 18.1% |
| No | 31.4% | 1.2% | 33.0% |
| **Current long COVID** |  |  |  |
| Overall | 6.3% | -7.5% | -1.7% |
| Age, year |  |  |  |
| 18–34 | 3.5% | -9.8% | -6.7% |
| 35–49 | -12.9% | -7.5% | -19.4% |
| 50–64 | 16.6% | -8.3% | 6.9% |
| ≥65 | 37.3% | -4.4% | 31.3% |
| Sex |  |  |  |
| Male | 8.7% | -3.0% | 5.4% |
| Female | 5.0% | -9.8% | -5.3% |
| Race/ethnicity |  |  |  |
| Non-Hispanic White | 3.8% | -4.9% | -1.3% |
| Non-Hispanic Black | 10.6% | 9.2% | 20.7% |
| Non-Hispanic other | 14.4% | -9.5% | 3.6% |
| Hispanic | 11.2% | -22.9% | -14.3% |
| Marital status |  |  |  |
| Married/unmarried couple | 5.7% | -9.1% | -3.9% |
| Single | 16.0% | -1.6% | 14.1% |
| Widowed/separated/divorced | 13.6% | -9.2% | 3.2% |
| Highest education level |  |  |  |
| Less than high school | 70.4% | -34.6% | 11.5% |
| High school graduate and some college | 1.2% | -7.4% | -6.3% |
| College graduate | 0.9% | 1.3% | 2.2% |
| Income |  |  |  |
| At or above poverty threshold | 4.0% | -3.9% | -0.1% |
| Below poverty threshold | 26.0% | -33.7% | -16.5% |
| Health insurance |  |  |  |
| Yes | 5.4% | -7.3% | -2.3% |
| No | 10.5% | -3.0% | 7.2% |

**Table S4. Prevalence of ever long COVID and current long COVID among US adults with prior COVID-19, 2022-2024**

|  | **Prevalence, % (95% CI)^a^** | | | **Changes across years, % (95% CI)^a^** | | | | | |
| --- | --- | --- | --- | --- | --- | --- | --- | --- | --- |
|  | **2022** | **2023** | **2024** | **2022 vs 2023^b^** | ***P* value^b^** | **2023 vs 2024^b^** | ***P* value^b^** | **2022 vs 2024^b^** | ***P* value^b^** |
| **Ever long COVID** |  |  |  |  |  |  |  |  |  |
| Long COVID reporters/participants, No. | 1,797/10,055 | 2,398/15,291 | 2,627/18,635 |  |  |  |  |  |  |
| Overall | 17.7 (16.8 to 18.6) | 15.1 (14.4 to 15.8) | 13.7 (13.1 to 14.4) | -2.6 (-3.7 to -1.5) | <0.001 | -1.3 (-2.2 to -0.4) | 0.003 | -3.9 (-5.0 to -2.8) | <0.001 |
| Age, year |  |  |  |  |  |  |  |  |  |
| 18–34 | 14.6 (13.2 to 16.2) | 13.3 (12.1 to 14.7) | 12.9 (11.7 to 14.2) | -1.3 (-3.3 to 0.7) | 0.202 | -0.4 (-2.2 to 1.3) | 0.622 | -1.7 (-3.7 to 0.2) | 0.080 |
| 35–49 | 20.1 (18.4 to 21.9) | 16.0 (14.6 to 17.4) | 14.9 (13.7 to 16.1) | -4.1 (-6.3 to -2.0) | <0.001 | -1.1 (-2.9 to 0.7) | 0.218 | -5.3 (-7.4 to -3.2) | <0.001 |
| 50–64 | 20.3 (18.4 to 22.3) | 16.9 (15.6 to 18.3) | 14.6 (13.4 to 15.8) | -3.4 (-5.7 to -1.0) | 0.006 | -2.3 (-4.1 to -0.6) | 0.008 | -5.7 (-7.9 to -3.5) | <0.001 |
| ≥65 | 16.1 (14.4 to 17.9) | 14.3 (13.1 to 15.7) | 12.4 (11.3 to 13.6) | -1.7 (-3.9 to 0.4) | 0.117 | -1.9 (-3.6 to -0.3) | 0.024 | -3.7 (-5.7 to -1.6) | 0.001 |
| Sex |  |  |  |  |  |  |  |  |  |
| Male | 13.7 (12.5 to 15.0) | 11.9 (11.1 to 12.9) | 11.3 (10.5 to 12.2) | -1.8 (-3.3 to -0.3) | 0.020 | -0.6 (-1.8 to 0.6) | 0.309 | -2.4 (-3.9 to -0.9) | 0.001 |
| Female | 21.2 (19.9 to 22.5) | 17.8 (16.9 to 18.8) | 15.9 (15.0 to 16.8) | -3.3 (-4.9 to -1.8) | <0.001 | -2.0 (-3.2 to -0.7) | 0.003 | -5.3 (-6.9 to -3.7) | <0.001 |
| Race/ethnicity^c^ |  |  |  |  |  |  |  |  |  |
| Non-Hispanic White | 18.2 (17.1 to 19.4) | 15.2 (14.3 to 16.1) | 14.0 (13.2 to 14.8) | -3.0 (-4.4 to -1.7) | <0.001 | -1.2 (-2.2 to -0.1) | 0.032 | -4.2 (-5.6 to -2.8) | <0.001 |
| Non-Hispanic Black | 15.5 (13.0 to 18.4) | 14.6 (12.5 to 17.0) | 14.4 (12.4 to 16.6) | -1.0 (-4.4 to 2.5) | 0.586 | -0.2 (-3.2 to 2.9) | 0.911 | -1.1 (-4.6 to 2.3) | 0.513 |
| Non-Hispanic other | 13.7 (11.0 to 16.9) | 11.6 (9.7 to 13.8) | 10.9 (9.2 to 12.9) | -2.1 (-5.5 to 1.3) | 0.218 | -0.7 (-3.3 to 1.9) | 0.613 | -2.8 (-6.0 to 0.4) | 0.085 |
| Hispanic | 18.6 (16.5 to 21.0) | 16.5 (14.9 to 18.4) | 13.9 (12.4 to 15.5) | -2.1 (-4.8 to 0.6) | 0.126 | -2.7 (-4.9 to -0.4) | 0.021 | -4.8 (-7.4 to -2.2) | <0.001 |
| Marital status |  |  |  |  |  |  |  |  |  |
| Married/unmarried couple | 18.0 (16.8 to 19.2) | 14.8 (14.0 to 15.7) | 13.7 (12.9 to 14.5) | -3.2 (-4.5 to -1.8) | <0.001 | -1.1 (-2.3 to 0.0) | 0.047 | -4.3 (-5.7 to -2.9) | <0.001 |
| Single | 14.1 (12.4 to 15.9) | 13.7 (12.3 to 15.2) | 12.6 (11.2 to 14.1) | -0.4 (-2.7 to 1.9) | 0.741 | -1.1 (-3.1 to 0.9) | 0.287 | -1.5 (-3.7 to 0.8) | 0.201 |
| Widowed/separated/divorced | 22.4 (20.1 to 24.8) | 19.6 (17.8 to 21.5) | 16.5 (15.0 to 18.2) | -2.8 (-5.7 to 0.2) | 0.066 | -3.0 (-5.4 to -0.7) | 0.010 | -5.8 (-8.6 to -3.0) | <0.001 |
| Highest education level |  |  |  |  |  |  |  |  |  |
| Less than high school | 18.7 (15.3 to 22.6) | 15.5 (13.1 to 18.3) | 15.5 (12.9 to 18.3) | -3.1 (-7.7 to 1.4) | 0.176 | -0.1 (-3.7 to 3.6) | 0.968 | -3.2 (-7.8 to 1.3) | 0.165 |
| High school graduate and some college | 19.0 (17.8 to 20.3) | 16.8 (15.7 to 17.8) | 15.2 (14.3 to 16.1) | -2.3 (-3.8 to -0.7) | 0.005 | -1.6 (-2.9 to -0.3) | 0.018 | -3.9 (-5.4 to -2.3) | <0.001 |
| College graduate | 15.2 (13.9 to 16.5) | 12.4 (11.5 to 13.4) | 11.4 (10.6 to 12.3) | -2.7 (-4.2 to -1.2) | <0.001 | -1.0 (-2.2 to 0.2) | 0.091 | -3.8 (-5.3 to -2.3) | <0.001 |
| Income |  |  |  |  |  |  |  |  |  |
| At or above poverty threshold | 17.4 (16.4 to 18.4) | 14.6 (13.8 to 15.3) | 13.4 (12.7 to 14.1) | -2.8 (-4.0 to -1.7) | <0.001 | -1.1 (-2.1 to -0.2) | 0.016 | -4.0 (-5.1 to -2.8) | <0.001 |
| Below poverty threshold | 20.6 (17.8 to 23.7) | 20.6 (17.9 to 23.5) | 17.6 (15.3 to 20.1) | 0.0 (-4.1 to 4.1) | 0.993 | -3.0 (-6.7 to 0.7) | 0.115 | -3.0 (-6.9 to 0.9) | 0.132 |
| Health insurance |  |  |  |  |  |  |  |  |  |
| Yes | 17.7 (16.8 to 18.7) | 14.9 (14.2 to 15.7) | 13.6 (12.9 to 14.3) | -2.8 (-3.9 to -1.6) | <0.001 | -1.3 (-2.2 to -0.4) | 0.005 | -4.1 (-5.2 to -2.9) | <0.001 |
| No | 17.2 (14.0 to 21.1) | 17.1 (14.2 to 20.5) | 15.6 (13.1 to 18.5) | -0.1 (-4.7 to 4.5) | 0.964 | -1.5 (-5.5 to 2.5) | 0.457 | -1.6 (-6.0 to 2.8) | 0.475 |
| **Current long COVID** |  |  |  |  |  |  |  |  |  |
| Long COVID reporters/participants, No. | 919/10,055 | 1,063/15,291 | 1,106/18,635 |  |  |  |  |  |  |
| Overall | 8.6 (8.0 to 9.3) | 6.5 (6.0 to 7.0) | 5.5 (5.1 to 5.9) | -2.1 (-2.9 to -1.3) | <0.001 | -1.0 (-1.6 to 0.4) | 0.002 | -3.1 (-3.8 to -2.3) | <0.001 |
| Age, year |  |  |  |  |  |  |  |  |  |
| 18–34 | 5.8 (4.9 to 6.9) | 4.6 (3.9 to 5.5) | 3.9 (3.3 to 4.7) | -1.2 (-2.4 to 0.1) | 0.061 | -0.7 (-1.7 to 0.3) | 0.163 | -1.9 (-3.1 to -0.7) | 0.002 |
| 35–49 | 10.6 (9.3 to 12.1) | 6.6 (5.7 to 7.8) | 5.7 (5.0 to 6.6) | -3.9 (-5.6 to -2.3) | <0.001 | -0.9 (-2.2 to 0.3) | 0.146 | -4.9 (-6.5 to -3.3) | <0.001 |
| 50–64 | 10.1 (8.9 to 11.6) | 8.0 (7.1 to 9.0) | 6.8 (5.9 to 7.8) | -2.1 (-3.8 to -0.5) | 0.012 | -1.2 (-2.5 to 0.1) | 0.069 | -3.4 (-5.0 to -1.7) | <0.001 |
| ≥65 | 8.9 (7.6 to 10.3) | 7.6 (6.7 to 8.6) | 6.3 (5.5 to 7.1) | -1.3 (-3.0 to 0.4) | 0.136 | -1.4 (-2.6 to -0.1) | 0.032 | -2.6 (-4.2 to -1.0) | 0.001 |
| Sex |  |  |  |  |  |  |  |  |  |
| Male | 6.0 (5.3 to 6.9) | 4.7 (4.1 to 5.3) | 4.2 (3.7 to 4.8) | -1.3 (-2.3 to -0.3) | 0.008 | -0.5 (-1.2 to 0.3) | 0.237 | -1.8 (-2.8 to -0.9) | <0.001 |
| Female | 10.9 (10.0 to 11.9) | 8.1 (7.4 to 8.8) | 6.6 (6.0 to 7.3) | -2.8 (-4.0 to -1.7) | <0.001 | -1.4 (-2.3 to -0.6) | 0.001 | -4.3 (-5.4 to -3.1) | <0.001 |
| Race/ethnicity^c^ |  |  |  |  |  |  |  |  |  |
| Non-Hispanic White | 9.5 (8.7 to 10.4) | 6.8 (6.2 to 9.3) | 5.8 (5.3 to 6.4) | -2.8 (-3.8 to -1.8) | <0.001 | -0.9 (-1.7 to -0.2) | 0.017 | -3.7 (-4.7 to -2.7) | <0.001 |
| Non-Hispanic Black | 7.0 (5.4 to 8.9) | 5.9 (4.6 to 7.5) | 6.2 (4.9 to 7.8) | -1.1 (-3.4 to 1.3) | 0.362 | 0.3 (-1.7 to 2.3) | 0.771 | -0.8 (-3.1 to 1.6) | 0.509 |
| Non-Hispanic other | 5.9 (4.2 to 8.2) | 4.7 (3.5 to 6.3) | 3.8 (2.7 to 5.2) | -1.2 (-3.5 to 1.1) | 0.311 | -0.9 (-2.7 to 0.8) | 0.298 | -2.1 (-4.5 to 0.2) | 0.072 |
| Hispanic | 7.6 (6.2 to 9.3) | 6.6 (5.5 to 8.0) | 4.9 (4.0 to 5.9) | -1.0 (-2.7 to 0.7) | 0.263 | -1.8 (-3.2 to -0.3) | 0.017 | -2.7 (-4.4 to -1.0) | 0.002 |
| Marital status |  |  |  |  |  |  |  |  |  |
| Married/unmarried couple | 9.0 (8.2 to 9.8) | 6.5 (5.9 to 7.2) | 5.5 (5.0 to 6.0) | -2.5 (-3.5 to -1.5) | <0.001 | -1.0 (-1.8 to -0.3) | 0.008 | -3.5 (-4.5 to -2.6) | <0.001 |
| Single | 5.3 (4.4 to 6.4) | 4.8 (4.0 to 5.8) | 4.4 (3.6 to 5.3) | -0.5 (-1.8 to 0.8) | 0.478 | -0.4 (-1.6 to 0.7) | 0.456 | -0.9 (-2.2 to 0.4) | 0.169 |
| Widowed/separated/divorced | 12.2 (10.5 to 14.1) | 9.9 (8.6 to 11.3) | 8.0 (7.0 to 9.3) | -2.3 (-4.5 to -0.1) | 0.044 | -1.8 (-3.6 to -0.1) | 0.040 | -4.1 (-6.4 to -1.9) | <0.001 |
| Highest education level |  |  |  |  |  |  |  |  |  |
| Less than high school | 6.8 (5.0 to 9.2) | 8.3 (6.5 to 10.7) | 5.6 (4.1 to 7.6) | 1.6 (-1.5 to 4.6) | 0.321 | -2.7 (-5.3 to -0.1) | 0.039 | -1.2 (-3.8 to 1.4) | 0.361 |
| High school graduate and some college | 9.7 (8.8 to 10.7) | 7.2 (6.5 to 8.0) | 6.2 (5.6 to 6.8) | -2.5 (-3.7 to -1.3) | <0.001 | -1.0 (-1.9 to -0.1) | 0.024 | -3.5 (-4.7 to -2.4) | <0.001 |
| College graduate | 7.3 (6.4 to 8.2) | 4.9 (4.4 to 5.6) | 4.5 (4.0 to 5.1) | -2.3 (-3.4 to -1.2) | <0.001 | -0.5 (-1.3 to 0.4) | 0.263 | -2.8 (-3.9 to -1.7) | <0.001 |
| Income |  |  |  |  |  |  |  |  |  |
| At or above poverty threshold | 8.5 (7.8 to 9.2) | 6.2 (5.7 to 6.8) | 5.5 (5.0 to 5.9) | -2.2 (-3.1 to -1.4) | <0.001 | -0.8 (-1.4 to -0.1) | 0.021 | -0.3 (-3.8 to -2.2) | <0.001 |
| Below poverty threshold | 9.9 (8.1 to 12.1) | 9.3 (7.5 to 11.4) | 6.0 (4.8 to 7.5) | -0.6 (-3.4 to 2.1) | 0.651 | -3.3 (-5.6 to -0.9) | 0.006 | -3.9 (-6.2 to -1.5) | 0.001 |
| Health insurance |  |  |  |  |  |  |  |  |  |
| Yes | 8.7 (8.1 to 9.4) | 6.5 (6.0 to 7.0) | 5.5 (5.1 to 6.0) | -2.2 (-3.0 to -1.4) | <0.001 | -1.0 (-1.6 to -0.3) | 0.003 | -3.2 (-4.0 to -2.4) | <0.001 |
| No | 7.1 (5.2 to 9.6) | 5.9 (4.2 to 8.2) | 5.2 (3.8 to 7.0) | -1.2 (-4.0 to 1.7) | 0.432 | -0.7 (-3.3 to 1.8) | 0.569 | -1.9 (-4.6 to 0.8) | 0.172 |

^a^ All estimates, except the numbers of participants and long COVID reporters, were weighted.

^b^ Linear regression was employed to estimate the absolute difference in prevalences.

^c^ Race and Hispanic ethnicity were self-reported and classified based on the 1997 Office of Management and Budget Standards.


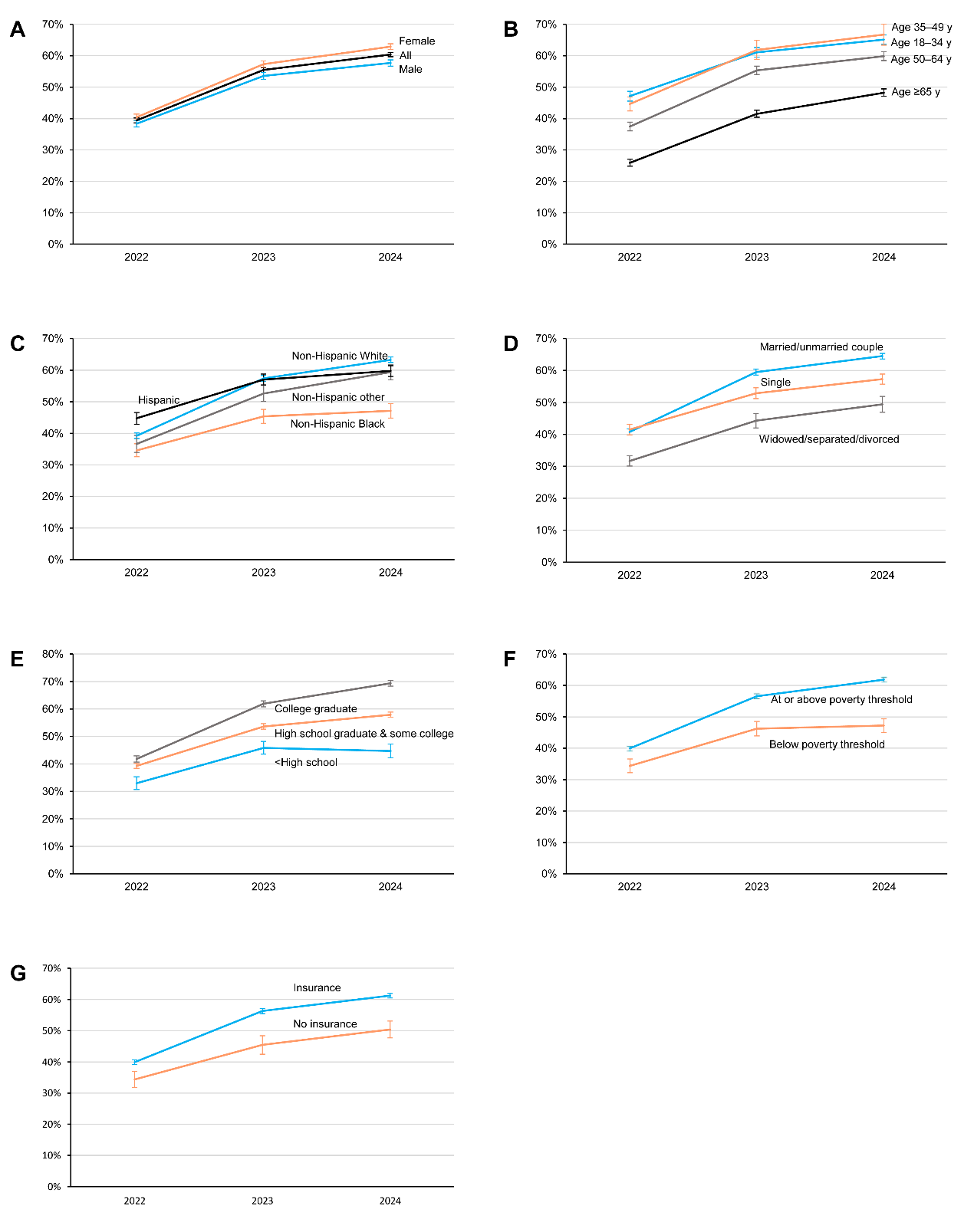


**Figure S1.** Cumulative infection rate of COVID-19 among overall US adults (A) and by sex (A), age (B), race/ethnicity (C), marital status (D), education (E), income (F), and insurance coverage (G) from 2022 to 2024. Error bars indicate 95% CIs.


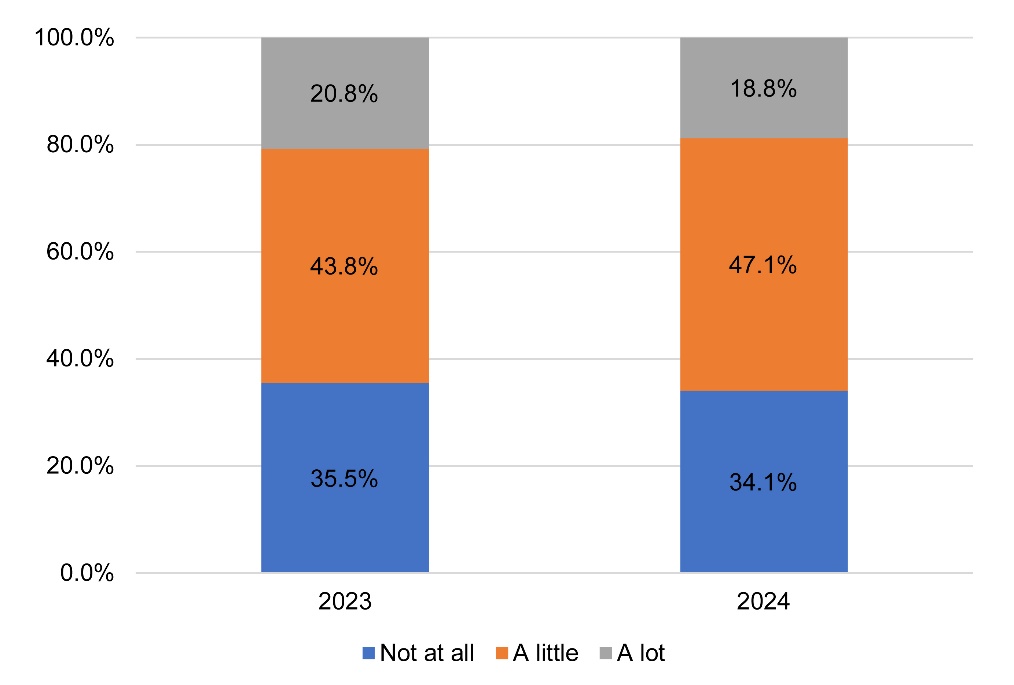


**Figure S2.** Daily activity limitation among US adults reporting current long COVID, 2023-2024


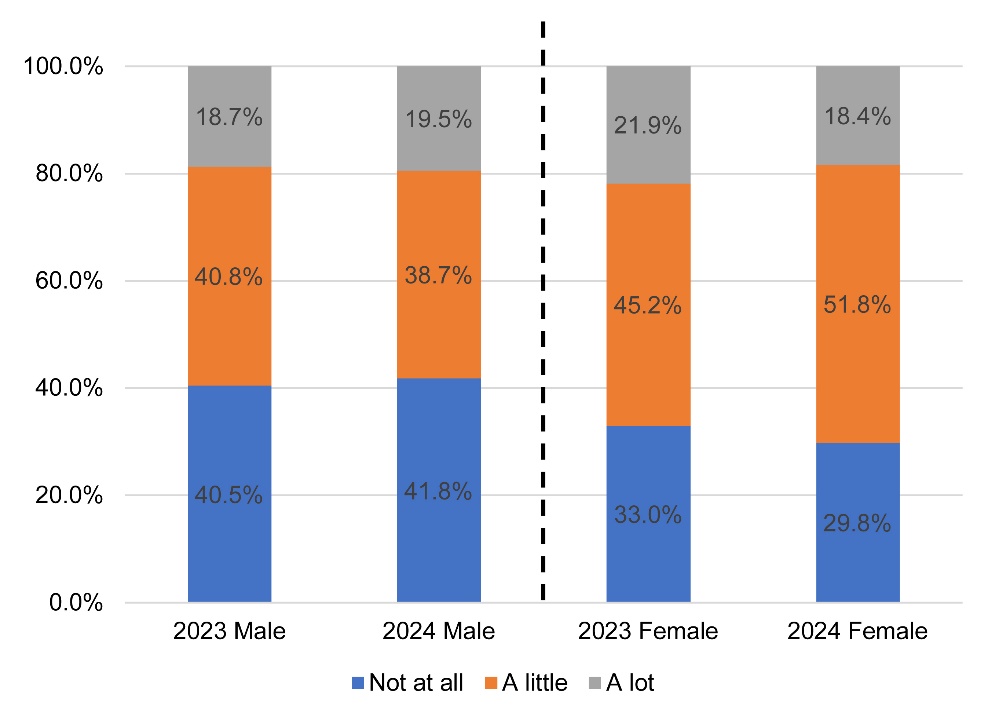


**Figure S3.** Daily activity limitation by sex among US adults reporting current long COVID, 2023-2024


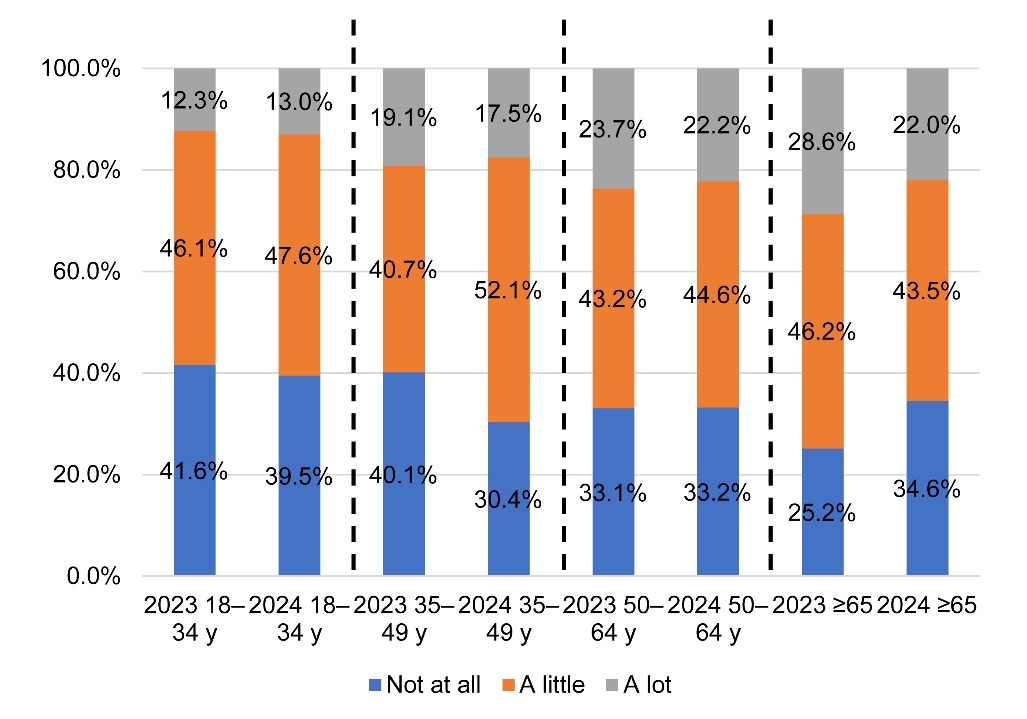


**Figure S4.** Daily activity limitation by age among US adults reporting current long COVID, 2023-2024


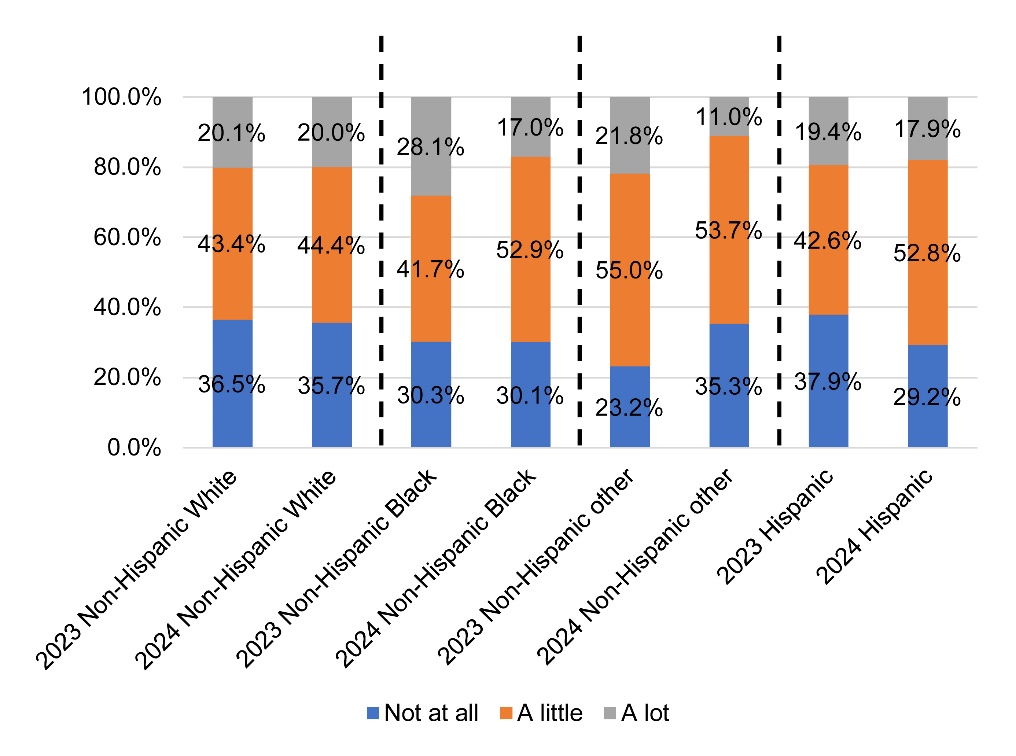


**Figure S5.** Daily activity limitation by race/ethnicity among US adults reporting current long COVID, 2023-2024


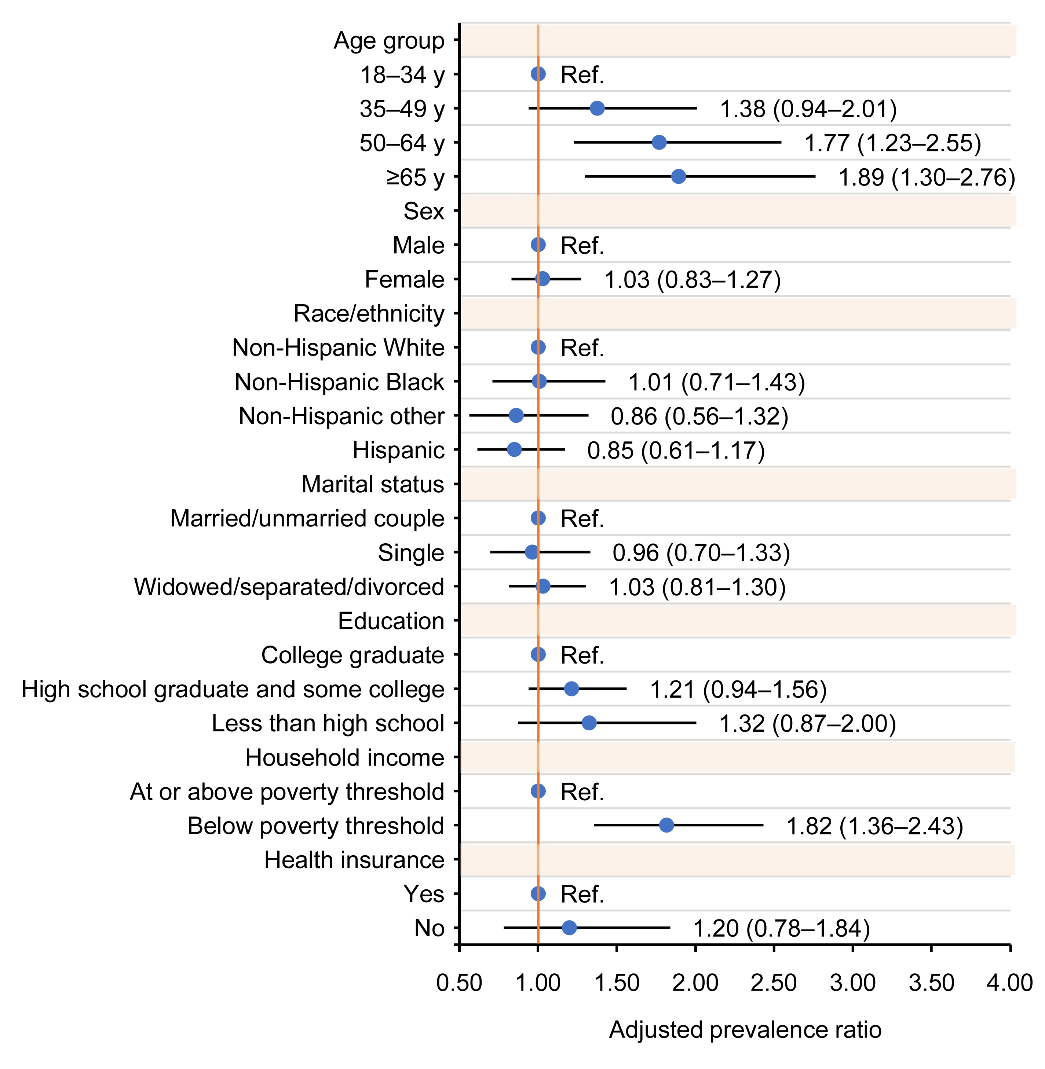


**Figure S6.** Factors associated with significant activity limitation among US adults with current long COVID. Multivariate Poisson regression with survey weights was used to adjust for age, sex, race/ethnicity, marital status, education, household income, and health insurance.
